# A New Bistable Switch Model of Alzheimer’s Disease Pathogenesis

**DOI:** 10.3390/ijms23137061

**Published:** 2022-06-25

**Authors:** Bruno Burlando, Serena Losacco, Viviana Villa, Ernesto Fedele, Roberta Ricciarelli

**Affiliations:** 1 Department of Pharmacy (DIFAR), University of Genoa, 16132 Genoa, Italy; bruno.pietro.burlando@unige.it (B.B.); serena.losacco.94@gmail.com (S.L.); 2 Department of Experimental Medicine (DIMES), University of Genoa, 16132 Genoa, Italy; viviana.villa@edu.unige.it; 3 IRCCS Ospedale Policlinico San Martino, 16132 Genoa, Italy

**Keywords:** amyloid-β, BACE1, cellular prion protein, cyclic nucleotides, bistable switch, feedback loop

## Abstract

We propose a model to explain the pathogenesis of Alzheimer’s disease (AD) based on the theory that any disease affecting a healthy organism originates from a bistable feedback loop that shifts the system from a physiological to a pathological condition. We focused on the known double inhibitory loop involving the cellular prion protein (PrPC) and the enzyme BACE1 that produces amyloid-beta (Aβ) peptides. BACE1 is inhibited by PrPC, but its inhibitory activity is lost when PrPC binds to Aβ oligomers (Aβo). Excessive Aβo formation would switch the loop to a pathogenic condition involving the Aβo-PrPC-mGluR5 complex, Fyn kinase activation, tau, and NMDAR phosphorylation, ultimately leading to neurodegeneration. Based on the emerging role of cyclic nucleotides in Aβ production, and thereby in synaptic plasticity and cognitive processes, cAMP and cGMP can be considered as modulatory factors capable of inducing the transition from a physiological steady state to a pathogenic one. This would imply that critical pharmacological targets for AD treatment lie within pathways that lead to an imbalance of cyclic nucleotides in neurons. If this hypothesis is confirmed, it will provide precise indications for the development of preventive or therapeutic treatments for the disease.

## 1. Introduction

Alzheimer’s disease (AD) is a neurodegenerative disorder that poses a huge public health problem as its prevalence is projected to triple worldwide by 2050. Two main mechanisms, possibly not mutually exclusive, have been classically proposed for the pathogenesis of AD, namely, the accumulation of amyloid-β (Aβ) plaques in the brain matrix and the hyperphosphorylation of tau protein forming intracellular neurofibrillary tangles. Despite decades of studies, however, the role of these protein aggregates is still elusive, the etiology of the disease remains unknown and, as a result, effective treatments are lacking [1]. One of the main drawbacks in identifying a pathogenic mechanism lies in the apparent multifactorial origin of the disease. Nevertheless, a unifying step is likely to occur during its initial course, and it is at this point in the development of the disease that therapeutic targets should be sought. The hypothesis we have developed precisely concerns this fundamental step and combines two paradigms: the first assumes that diseases arise from the dynamics of positive feedback loops [2], and the second attributes a central pathogenic role to the malfunctioning of cyclic nucleotide signaling in cognitive processes [3].

## 2. BACE1, PrPC and the Positive Feedback Loop

According to the Systems and Control Theory [4], various biological processes, especially irreversible ones (e.g., mitosis), can be modeled by positive feedback loops, which are closed chains of interactions that have no inhibitory steps or an even number of them. The behavior of these loops can give rise to multistable systems that are capable, at least in principle, of triggering the transition from a healthy to a sick condition, with profound repercussions in pathophysiology [5].

**Figure 1:**
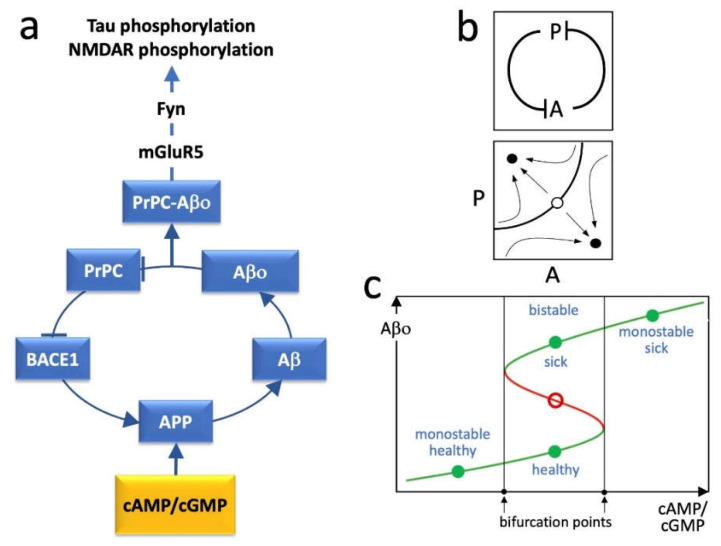
(**a**) Schematic graph of the proposed AD pathogenic loop showing the involved molecular agents. Cyclic nucleotides act as external elements providing an input to the loop in correspondence of its APP component. Arrow-ended lines = activation; bar-ended lines = inhibition; APP = amyloid precursor protein; Aβ = amyloid-β; Aβo = amyloid-β olygomers; BACE1 = β-secretase 1; Fyn = Fyn kinase; mGluR5 = metabotropic glutamate receptor 5; PrPC = cellular prion protein. (**b**) Top panel: simplified diagram of the loop system reported in (**a**), where A represents the complex of agents, from BACE1 to Aβo, and P represents PrPC. Bottom panel: bidimensional phase portrait in the P/A plane showing hypothetical attraction basins of stable equilibrium points (filled circles) and an unstable equilibrium point (open circle). (**c**) Curve of the steady states (equilibrium points) of Aβo, representing a loop variable, as a function of cAMP/cGMP stimulus. The latter acts as a bifurcation parameter able to shift the loop dynamics from monostability to bistability. In the bistable zone, the system admits two stable equilibrium points and an unstable one for each cAMP/cGMP input. Green curve branches and filled circles = stable equilibrium points; red curve branch and open circle = unstable equilibrium points.

Based on the huge amount of data in the literature, two possible key elements in the pathogenesis of AD can be identified, namely, beta-secretase 1 (BACE1) and the cellular prion protein (PrPC). BACE1 is an enzyme required to produce amyloid-β (Aβ), which, after oligomerization, binds to PrPC [6]. On the other hand, PrPC inhibits the activity of BACE1 [7], but this effect is disturbed by the Aβ oligomers (Aβo) bound to the protein [6] (Figure 1a). Hence, the system is structurally organized as a double inhibitory loop involving the inhibitory activity of BACE1 on PrPC, through Aβo formation, and the inhibitory activity of Aβo-free PrPC on BACE1 (Figure 1b). Having an even number of inhibitory steps, the system behaves like a positive feedback loop with multistable dynamics and at least two stable equilibrium points [8]. We believe that these equilibrium points reflect, respectively, a physiological steady state with low Aβ production and prevalent Aβo-free PrPC, and a pathogenic steady state with high Aβ production and prevalent Aβo-bound PrPC (Figure 1c).

In neurons, Aβ is positively modulated by the cyclic nucleotides cAMP and cGMP. Specifically, cAMP promotes the production of Aβ by stimulating the expression of the amyloid precursor protein (APP) [9,10], while cGMP favors the amyloidogenic processing of APP by BACE1 [11]. These are physiological events essential for the expression of hippocampal long-term potentiation (LTP) and for memory formation/consolidation. However, an excessive increase in Aβ would lead to an increase in Aβo bound to PrPC, thus switching the loop to the pathogenic steady state, with low inhibitory activity of PrPC on BACE1 (Figure 1c).

The signal transduction downstream of Aβo-PrPC complexes involves mGluR5 and the Fyn tyrosine kinase [12,13] (Figure 1a). Notably, Fyn hyperactivation is followed by a series of events, including tau and NMDAR phosphorylation, synaptotoxicity, and finally neurodegeneration [14].

## 3. Loop Dynamics

The dynamics of the positive feedback loop depicted in Figure 1a can be modeled by a system of ordinary differential equations (ODEs). Since biological networks can be conveniently considered as aggregates of monotone subsystems [15], the loop can be reduced to a sequence of two inhibitory steps (Figure 1b) and its dynamics can be represented by a system of two ODEs yielding the rate of change of the functional agents. Each functional agent *X* is assumed to undergo spontaneous inactivation and evolves over time with the time constant $\tau_{X}$. The ODE system, in a schematic form, is the following:

(1) $$\tau_{A}\dot{A}+A=g\left(P\right)$$

(2) $$\tau_{P}\dot{P}+P=g\left(A\right)\;$$

where $\dot{A}$ and $\dot{P}$ are the time derivatives of *A* and *P*, respectively, *P* represents PrPC, and *A* is the complex of loop agents from BACE1 to Aβo. The two *g*(⋅) functions do not need to have the same form, but, for instance, can have the form of decreasing Hill-type expressions:

(3) $$g\left(x\right)=\frac{\gamma }{\delta^{h}+x^{h}}$$

with *h* being the Hill coefficient, and γ and δ being positive real parameters. A wide complex of data indicates that Hill functions can be used to model biological dose–response interactions [16]. In the ODEs, the parameters that influence the maximal value attainable by *A*, or its decrease for increasing values of *P*, represent the stimulatory effect of cAMP or cGMP on the levels of Aβ. These parameters are expected to act as bifurcation parameters able to convert the system from monostable to bistable and vice versa (Figure 1c).

It must be noted that this kind of model has already been applied to a series of pathogenic mechanisms, a few representative examples of which include cancer [16], immunological disorders [5], neurological problems [17], and neurodegenerative diseases [18]. The mathematical evaluation of these loops can be used to identify the bifurcation parameters that drive the transition from monostability to multistability, thus determining the switch from a “physiological” to a “pathological” steady state condition [8]. In this perspective, the biological correspondents of the bifurcation parameters would represent the best therapeutic targets.

## 4. Conclusions

Our study starts from the evidence that biomedical research on AD pathophysiology is facing serious drawbacks in attempting to provide a tenable pathogenic mechanism. We believe that such a disappointing state of the art can only be overcome by a full reconsideration of the biological context of the disease. The pathogenesis model we propose considers two completely innovative aspects in AD research, namely, the use of a multistationary positive loop and the central role of cyclic nucleotides. These two elements are closely related, since cyclic nucleotides play the role of loop bifurcation parameters and, therefore, their imbalance will tend to shift the system from the steady state of low Aβ production to the steady state of high Aβ production, high levels of the PrPC-Aβo complex and neurodegenerative consequences.

The model would, therefore, function as a bottleneck for different, or multifactorial, AD causes. Considering that (i) cAMP and cGMP are involved in the different phases of LTP correlated with short- and long-term memory, and (ii) consistent evidence demonstrates that Aβ plays a critical role in LTP expression and memory consolidation, where it seems to act as a common effector of cAMP/cGMP signaling, it can be speculated that, under normal conditions, cyclic nucleotides favor synaptic plasticity and memory by triggering the physiological production of Aβ. From this point of view, overstimulation of this physiological process could lead to different pathological scenarios. An excess of cyclic nucleotides, for example, would abnormally increase Aβ levels, thus favoring its aggregation and limiting the availability of functionally active soluble oligomers, or alternatively, it could exhaust the ability of neurons to produce functional Aβ, similar to what happens with insulin in a pancreas undergoing chronic hyperglycemic stimulation.

At the same time, the excessive formation of Aβo-PrPC complexes would stimulate the hyperactivation of Fyn [12,13,14] and possibly other kinases, leading to the hyperphosphorylation of tau and well-known pathogenic consequences. In support of this, our recent studies have highlighted the role of both cGMP and cAMP in tau phosphorylation [19,20].

In conclusion, our model provides a unifying explanation for a large body of evidence accumulated over years of AD studies. However, while this is a strong indication of validity, distinct proof-of-concept experiments remain necessary. One of the most critical aspects of the analysis is the derivation of a suitable set of ODEs that represent the interactions between the variables of the system. The ODE formulation will have to be achieved from literature data and through the design of targeted experiments (e.g., see [21]). By finely manipulating the concentration of cyclic nucleotides within neuronal cells, it will have to be shown that a positive feedback loop depending on cyclic nucleotides and PrPC is able to regulate Aβ production through a bistable system. Furthermore, under cyclic nucleotide dysregulation, the bistable switch will have to induce high levels of Aβo, followed, for example, by hyperphosphorylation of tau, as a marker of the pathophysiological transition towards the disease. Subsequently, the loop system will have to be validated using animal models, to demonstrate that cyclic nucleotide manipulation can influence the onset/regression of the disease in vivo.

The main assumption and strength of pathophysiological models based on multistable systems is that the bifurcation parameters derivable from mathematical analysis represent the biological factors that must be addressed in the attempt to predictably manipulate the system. This means that if the possibility of arresting or reverting a pathological condition exists, however recalcitrant to clinical remedies, the goal is to identify and modulate these factors. Although further studies are needed, the model proposed here already indicates that the two cyclic nucleotides could act as bifurcation parameters, suggesting that critical therapeutic targets should be sought within their metabolic pathways.

## Data Availability

Not applicable.
